# *Prosopis juliflora* (Sw.), DC induces apoptosis and cell cycle arrest in triple negative breast cancer cells: *in vitro* and *in vivo* investigations

**DOI:** 10.18632/oncotarget.25717

**Published:** 2018-07-13

**Authors:** Bhimashankar Gurushidhappa Utage, Milind Shivajirao Patole, Punam Vasudeo Nagvenkar, Sonali Shankar Kamble, Rajesh Nivarti Gacche

**Affiliations:** ^1^ National Centre for Cell Science, NCCS Complex, Pune, 411007, MS, India; ^2^ School of Life Sciences, S.R.T.M. University, Nanded, 4316069, MS, India; ^3^ Department of Biotechnology, Savitribai Phule Pune University, Pune, 411007, MS, India

**Keywords:** *P. juliflora*, triple negative breast cancer, tumor inhibition, apoptosis, ROS

## Abstract

Plant originated drugs/formulations are extensively prescribed by the physicians as a complementary therapy for treating various human ailments including cancer. In this study *Prosopis juliflora* leaves methanol extract was prepared and exposed to human breast cancer cell lines i.e. MDA-MB-231 and MCF-7 and human keratinocytes HaCaT as a representative of normal cells. Initially, a series of *in vitro* experiments like cell proliferation, migration, colony formation, cell cycle arrest and inhibition of angiogenesis. After confirmation of the efficient and selective activity against triple negative breast cancer cell line, we further evaluated the possible mechanism of inducing cell death and experiments like detection of reactive oxygen species, caspases and poly (ADP-ribose) polymerase cleavage study and Annexin V assay were performed. We also evaluated *in vivo* anti tumorigenic activity of the *P. juliflora* leaves by using 4T1 cells (a triple negative mouse origin breast cancer cell line) and BALB/c xenograft mouse model. *In vitro* experiments revealed that methanol extract of *Prosopis juliflora* leaves possess impressive anti-breast cancer activity more specifically against triple negative breast cancer cells, while the *in vivo* studies demonstrated that *P. juliflora* leaves extract significantly suppressed the 4T1 induced tumor growth. Present investigations clearly focus the significance of *P. juliflora* as an important resource for finding novel leads against triple negative breast cancer. The results may also act as a ready reference towards developing *P. juliflora* based formulation as an alternative and complementary medicine for the management of breast cancer.

## INTRODUCTION

The media center of WHO in Feb 2017 [1] states that breast cancer mortality ranks fifth amongst the cancer related deaths in world, moreover the breast cancer related mortality is increasing both in the developed and the developing countries. More specifically, breast cancer is the most common cancer diagnosed among US women and has been identified as the second leading cause of cancer mortality among women after lung cancer. It has been estimated that over 266,120 new cases of invasive breast cancer will be diagnosed among women and over 2,550 cases will be diagnosed in men in 2018. Moreover, there forecast alarms that over 63,960 cases of *in situ* breast carcinoma will be diagnosed in women. It is speculated that approximately 40,920 women and 480 men are expected to succumb to death from breast cancer in 2018 [2]. Besides the several factors of causation, inherited mutations in BRCA1 and BRCA2 genes have been accounted for 15%-20% of all familial breast cancers [3]. Based on the routinely evaluated biological markers molecular subtypes of breast cancers have been classified as presence or absence of hormone (estrogen or progesterone) receptors (HR^+^/HR^-^) and presence or absence of excess levels of human epidermal growth factor receptor 2 (HER2^+^/HER2^-^). Besides this, four main molecular subtypes such as Luminal A (HR^+^/HER2^-^) (accounting 71%), triple negative breast cancer: TNBC (HR^-^/HER2^-^) (accounting 12%), Luminal B (HR^+^/HER2^+^) (accenting 12%) and HER2-enriched (HR^-^/HER2^+^) (accounting 5%) have been described. As compared to hormone dependent breast cancer, TNBC are difficult to treat owing to non-expression of ER, PR or HER2 receptors and the currently available anti-breast cancer hormone therapies target one of the three receptors [4]. Also, In India TNBC cases prevalence is higher than the other countries in the world [5].

In the current state of the art, surgery (including prophylactic), radiotherapy and chemotherapy are the major three treatment regimens employed for the management of breast cancer. Besides the impressive effects of the chemotherapeutic drugs, there are several side effects which limit the efficacy and usage of presently available chemotherapy drugs. The most notable side effects of chemotherapeutic drugs include hot flashes, nausea, and fatigue. Premenopausal women using tamoxifen can also experience adverse effects like blood clots, risk of endometrial cancer, and adversities in menstrual cycle [6]. Last two decades research in cancer biology has brought a paradigm shift in understanding the pathophysiology of cancer progression. In the current scenario, cancer progression in general and breast cancer in particular is being studied in context with evolving ecosystem in concert with evolving intratumor heterogeneity. Cellular heterogeneity in the tumor has been identified a major culprit for fostering tumor evolution and perhaps in the contemporary issues of managing cancer treatment it coming up as a major challenge in the management of breast cancer. Sizable preclinical and clinical evidence has established the fact that tumor heterogeneity happens to be one of the significant factors imparting drug resistance in breast cancer [7]. Therefore in the midst of heterogeneity driven drug resistance, there is need to identify novel leads against breast cancer, which perhaps circumvent the emerging drug resistance and evolving tumor heterogeneity.

Since, ancient time natural products and drug discovery, especially ‘medicinal plants’ has remained a significant hope for the discovery of novel drugs against a variety of human ailments. More encouraging fact is that the physicians across the world have started prescribing the plant based traditional (otherwise novel formulations) drugs as a complementary and alternative medicinal (CAM) therapy which either complements the efficacy of existing drugs or works as a standalone therapeutic approach for the treatment of variety of human ailments including breast cancer. Being a beneficial cocktail of conventional and CAM, a concept of ‘integrative oncology’ is coming up with a possible inclusion of plant-based drugs, reverse pharmacology and holistic approach for the effective management of a variety of human cancers including breast cancer. The circumstantial literature accumulating in the recent past clearly advocates the significance of evidence-based integrative approach for the management of breast cancer [8]. Crude extracts of *Allium sativum*, *Curcuma longa*, *Echinacea*, *Arctiumlappa*, *Panax ginseng*, *Camellia sinensis*, extracts of Flax seed, extracts and decoctions of *Withania somnifera*, *Amoora rohituka*, *Dysoxylum binectariferum* and *Vaccinium macrocarpon* are some of the representative herbal agents which are traditionally used for the treatment of breast cancer [9]. The plant *Prosopis juliflora* (*P. juliflora*) selected in the present study is mostly used for providing house wood/firewood, livestock feed and interestingly the ‘pods’ contains large amounts of sugar therefore, in the most part of the world it is regularly used in the human diet as a pod syrup, coffee substitute, flour, beverage, fermented beverage and baked products as well [10, 11]. Traditionally *P. juliflora* is also used as a folk remedy for treating diseases like flu, cold, excrescences, inflammation, measles, diarrhea, dysentery, sore throat and in healing of wounds [12, 13]. A review by Khandelwal et al. (2015) described different aspects of *P. Juliflora* including the major pharmacological attributes like antagonistic effect, anti-bacterial, antioxidant and anticancer activities [14]. More precisely, the anti-cancer activity of *P. Juliflora* leaf alkaloids have been shown to be preferentially cytotoxic against human T-cell leukemia (Molt-4) cells in a time and dose-dependent manner under *in vitro* conditions and interestingly the *P. juliflora* leaf alkaloids have not demonstrated genotoxicity [15]. In general discourse, it has been proved to a greater extent that plants experiencing extreme stress environment are more efficient in producing high amounts of stress defensive secondary metabolites, which perhaps contain the bioactive molecules for the treatment of human diseases [16]. With this circumstantial background literature and inspired by the extreme xerophytic sustainability of *P. juliflora*, we planned a study for investigating the anti-breast cancer potential of *Prosopis juliflora* leaves methanol extract (PJLME) against MDA-MB-231 and MCF-7 breast cancer cells by employing a variety of *in vitro* and *in vivo* experimental settings.

## RESULTS

### Effect of PJLME on MDA-MB-231, MCF-7 and HaCaT cell proliferation

**Figure 1:**
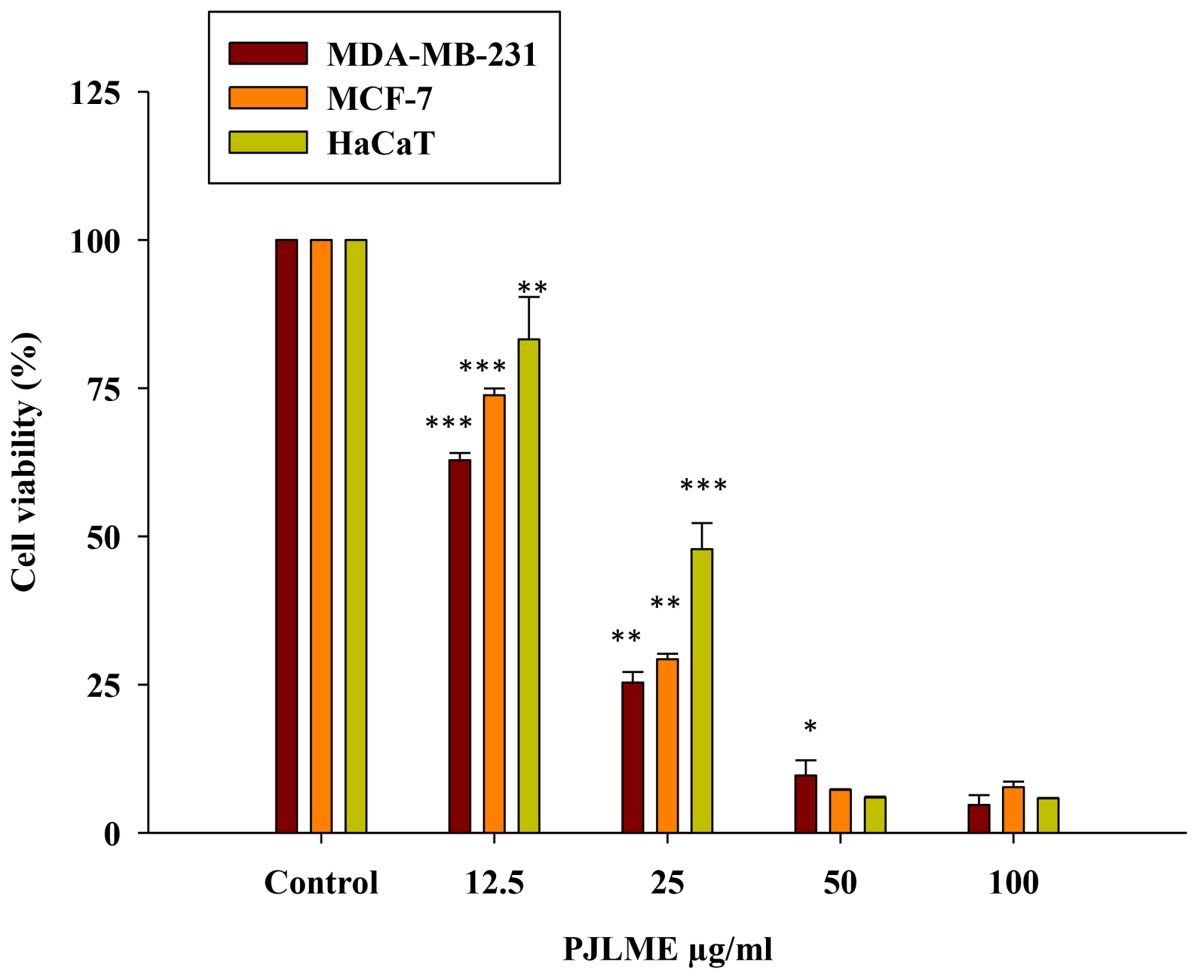
Effect of PJLME on viability of MDA-MB-231, MCF-7 breast cancer cells and normal HaCaT cells MTT cell viability assay was performed for assessing the effect of PJLME on selected cells. Results are expressed as the mean ± standard deviation of three independent experiments. ^*^P<0.05, ^**^P<0.01, ^***^P<0.001 vs. control (DMSO 0.1%).

For the evaluation of antiproliferative activity of PJLME against tumorigenic breast cancer cell lines (MDA-MB-231, MCF-7) and non-tumorigenic keratinocytes (HaCaT), we initially performed ‘MTT Assay’ with the different concentrations (12.5, 25, 50 and 100μg/mL). The assay involves conversion of MTT tetrazolium salts to a colored formazan and this product formed is directly proportional to the number of metabolically active cells (mitochondria) and thus absorbance measured is indirectly related with the number of proliferating cells. In this study two human breast cancer-specific cell lines were used, one of them was TNBC cell line MDA-MB-231 (ER, PR and HER’s-2 receptor negative) and other was estrogen receptor positive MCF-7 cell line. As a representative of normal cells, a HaCaT (human keratinocytes) cell line was used. All these human cell lines were treated with different concentrations of PJLME up to72 hours. The result of MTT assay clearly showed that PJLME reduced percent cell survival in a time and dose-dependent manner (Figure 1). It was found that MDA-MB-231 cells were found to be more sensitive towards PJLME treatment (IC50 16.8 μg/mL) as compared to treatment with MCF-7 cells (19.4 μg/mL). Interestingly, normal HaCaT cells were found to be less sensitive towards PJLME treatment (IC50 24.1μg/mL) as compared to treatment with MDA-MB-231 and MCF-7 breast cancer cells.

### Effect of PJLME on morphology of MDA-MB-231, MCF-7 and HaCaT cells

**Figure 2:**
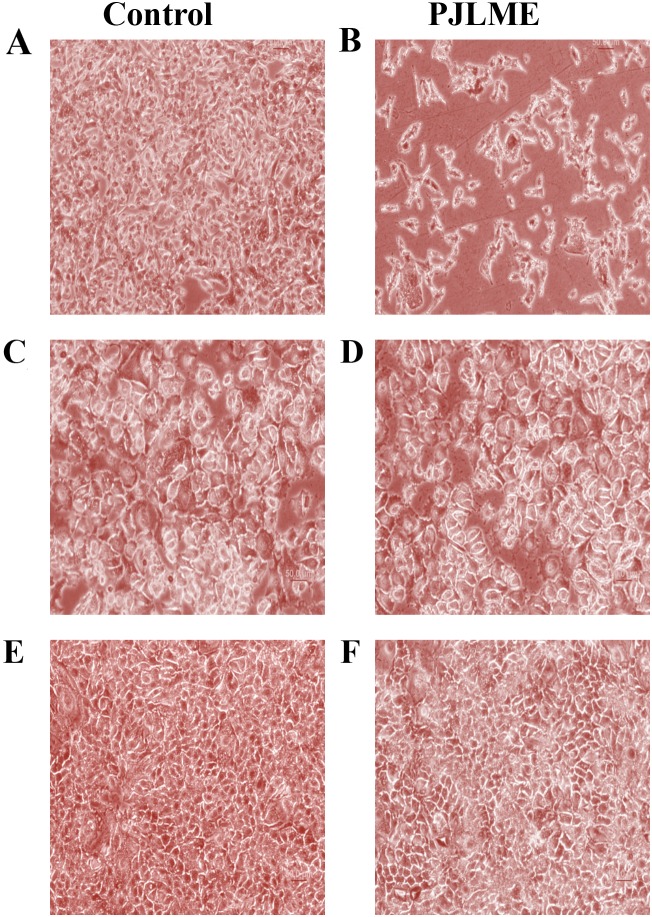
Effect of PJLME on morphology of MDA-MB-231, MCF-7 and HaCaT cells **(A)** Control MDA-MB-231cells **(B)** PJLME treated MDA-MB-231 Cells **(C)** Control MCF-7 cells **(D)** PJLME treated MCF-7 cells **(E)** Control HaCaT cells **(F)** PJLME treated HaCaT cells. The cells were treated with PJLME (16.8 μg/mL) and vehicle control (DMSO 0.1% ) for 72h and the images were digitizedat10X magnification using an inverted Nikon microscope equipped with a digital camera.

In order to assess the effect of PJLME on the morphology of the selected cells, the cells were treated with PJLME (16.8 μg/mL; an IC_50_ value of MDA-MB-231 cell line). After 72 hours of treatment, the cellular morphology of the PJLME treated cells was observed under phase contrast microscope. The digitized images of the PJLME treated cells (Figure 2) revealed the abnormal cell morphology as compared to the cells treated with DMSO as a vehicle. The morphological changes were also observed in the form of reduction in cell number and volume, rounding off, shrinkage and appearance of floating or dead cells. It was observed that adverse morphological effects were more predominant in MDA-MB-231 cells. It was also observed that PJLME treatment had very marginal adverse effect on the morphology of MCF-7 and HaCaT cells (Figure 2). The adverse morphological changes were only apparent (images not shown) when these cells were treated with the higher concentration (> 100 μg/mL) of PJLME.

### Effect of PJLME on cell migration (Scratch Assay)

**Figure 3:**
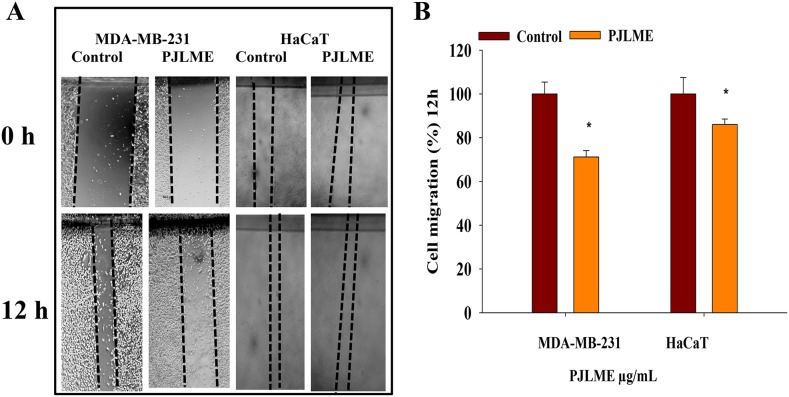
**(A)** Effect of PJLME on cell migration. For evaluation of the migration inhibitory properties of the PJLME, scratch assay was performed on the confluent monolayer's of MDA-MB-231 and HaCaT cells. (A) Images of the vehicle control (DMSO 0.1%) and PJLME treated (16.8 μg/mL) cells at 0 and 12 hours of cell migration. **(B)** Migration (%) of vehicle control and PJLME treated cells. ^*^values that were significantly different (^*^p < 0.05) from the DMSO control.

Cell migration assays are usually studied to forecast the anti-metastatic potentials of the drug candidates. Series of herbal extracts have been documented for their inhibitory effects on cell migration, which perhaps can be attributed with their anti-metastatic potentials [17]. For assessing the cell migration inhibitory potential of PJLME, a scratch assay was performed, wherein the selected cell lines were treated with PJLME (16.8 μg/mL) and the cell migration potential was quantified. The results shown in Figure 3A and 3B) clearly demonstrate that PJLME has impressive inhibitory effect on migration of the triple negative MDA-MB-231 cells (71%) as compared to the migration of DMSO control (100%). PJLME has almost no effect on the migration ability of normal HaCaT cells (85%) revealing that the contents of *P. juliflora* discriminate between breast cancer cells and normal cells.

### Effect of PJLME on colony formation (Clonogenic Assay)

**Figure 4:**
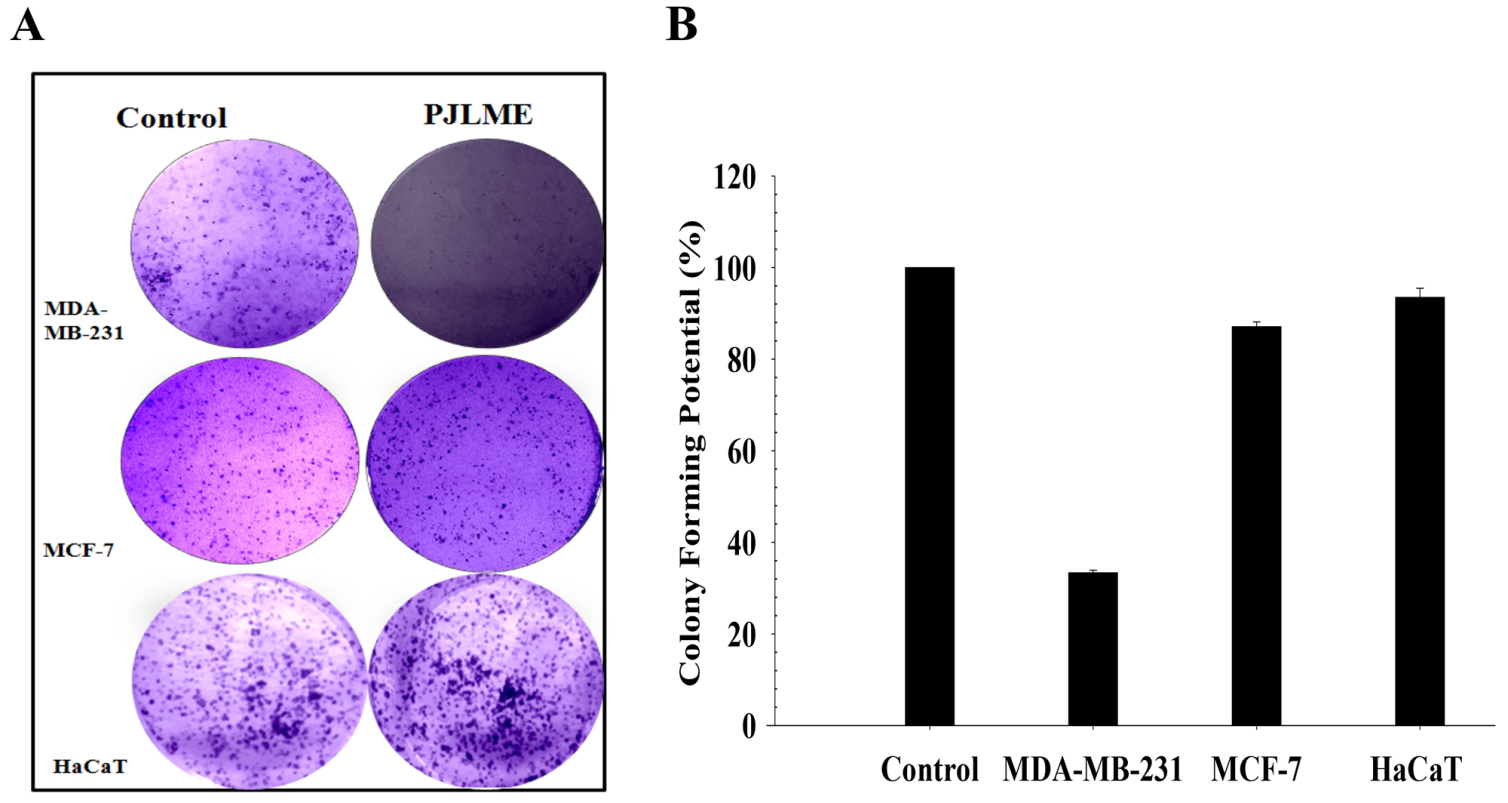
**(A)** Effect of PJLME on colony formation. For assessing the effect of PJLME on colony forming potential, clonogenic assay was performed. Selected cell lines were treated with PJLME (16.8 μg/mL) and vehicle control (DMSO 0.1%) for 72h and re-cultured for next seven days. Stained cells were observed & colonies were counted using phase contrast microscope (Olympus, Tokyo, Japan) equipped with digital camera. (A) Images of the colonies formed in treated and control sets. **(B)** Statistical analysis of the clonogenic assay.

Efficiency of the PJLME to inhibit the regenerative ability (unlimited cell divisions: a hallmark of cancer) of the treated cells was evaluated using clonogenic assay. Breast cancer cells such as MDA-MB-231, MCF-7 and non-tumorigenic HaCaT cells were treated with the PJLME (16.8 μg/mL) up to 72 hrs. After treatment duration, the cells were reseeded with the regular growth medium containing 10% FBS for 7-12 days. Microphotograph & statistical analysis (Figure 4A and 4B) of the treated cells clearly indicated that PJLME treatment significantly inhibited the colony forming or regenerative ability of MDA-MB-231 cells. The results also confirmed that the inhibition of the colony formation effect of PJLME was not significant with MCF-7 cells, once again it suggest that the phytochemical contents of *P. juliflora* seems to be more specific towards inhibiting the growth and related physiological functions of triple negative MDA-MB-231 cells. Interestingly, the same was observed with non-tumorigenic normal HaCaT (keratinocytes) cells.

### Detection of intracellular reactive oxygen species

Reactive oxygen species (ROS) have been described as highly reactive free radical's which indiscriminately reacts with variety of biological molecules like DNA, proteins, lipids etc. When ROS concentration exceeds in the cells, they adversely affect the cell physiology leading to cell death [18, 19].

**Figure 5:**
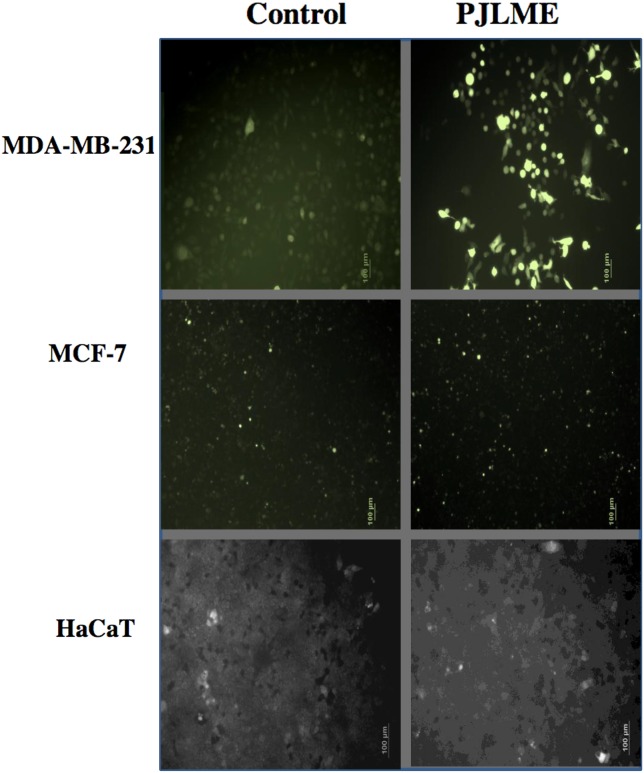
Detection of PJLME induced intracellular reactive oxygen species (ROS) For detection of ROS, the cells were treated with PJLME (16.8 μg/mL) for 72h and were subsequently exposed to DCFH-DA (10 μM) dye. The results were compared with control set (DMSO 0.1%). Intracellular ROS generation was observed and images were captured by using fluorescence microscope (Olympus, Tokyo, Japan).

For detecting the PJLME induced intracellular ROS generation, the selected cell lines (MDA-MB-231, MCF-7 and HaCaT) were treated with PJLME (16.8 μg/mL) up to 72h. After the treatment, the cells were stained with DCFH-DA and were observed under fluorescence microscope (Olympus, Tokyo, Japan). Resulting digitized images (Figure 5) clearly demonstrated the excessive ROS production in the cytoplasm of the MDA-MB-231 cells, while non-significant ROS generation was observed in MCF-7 cells. Of note, PJLME treatment with HaCaT cells could not induce considerable ROS generation as compared to MDA-MB-231 cells. ROS induced cell death might be one of the mechanism of cell death, and perhaps a reason for differential cell viability profile of the selected cells. The results of ROS generation also reiterate the MDA-MB-231 cell specific activity of PJLME.

### Detection of apoptosis using nuclear fragmentation assay

**Figure 6:**
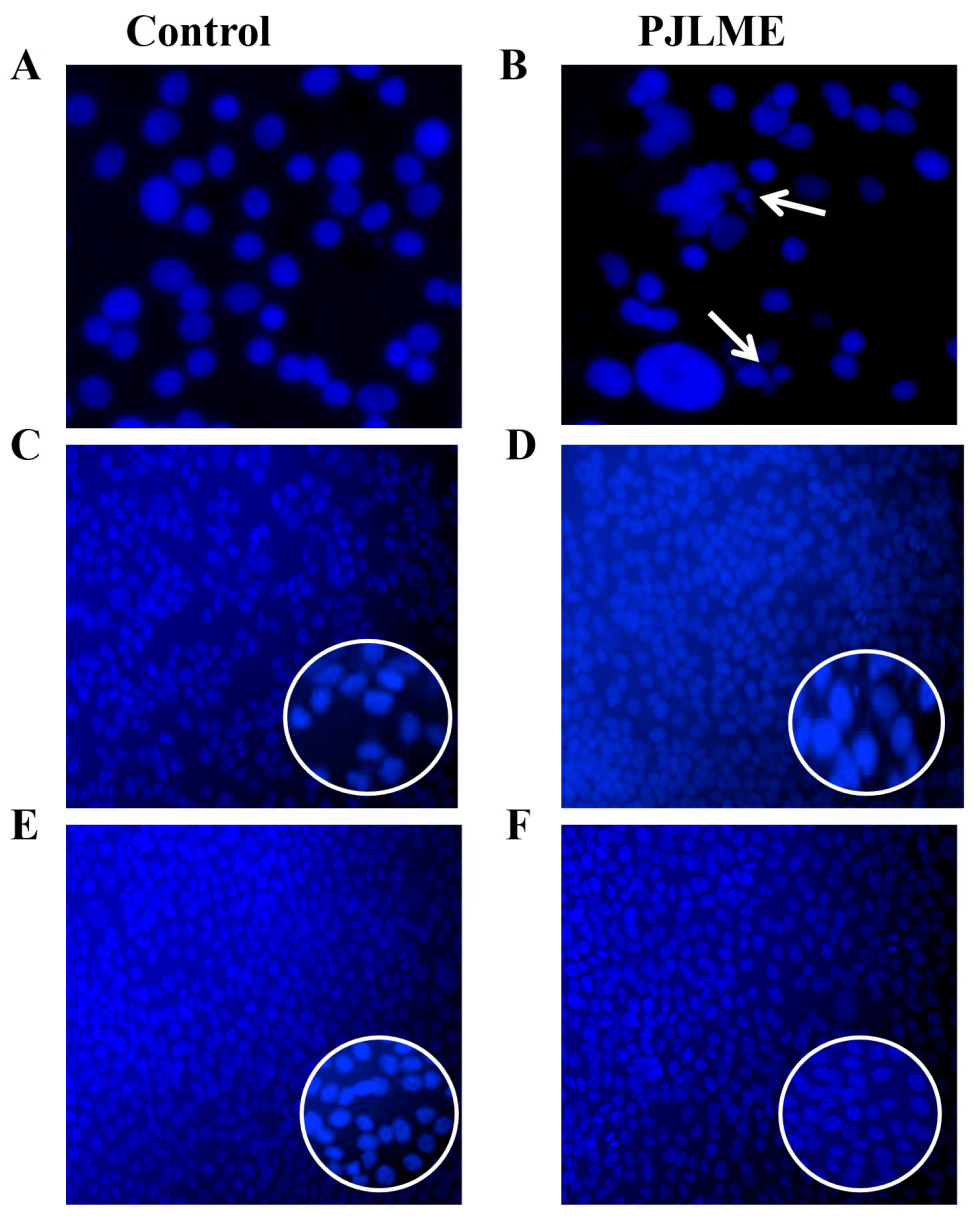
Detection of PJLME induced nuclear fragmentation Representative digitized images are of PJLME (16.8 μg/mL) and vehicle control (DMSO 0.1%) treated cells for 72h. **(A)** and **(B)** MDA-MB-231cells, **(C)** and **(D)** MCF-7 cells, **(E)** and **(F)** HaCaT cells. Photographs were taken at 10X magnification using an inverted Nikon microscope equipped with a digital camera.

One of the sign of the apoptosis is a ‘pyknosis’ which causes chromatin condensation and fragmentation [20]. The selected cells were exposed to PJLME (16.8 μg/mL) up to 72h followed by staining with Hoechst 33258 stain. The observations of the stained cells revealed that the cells treated with vehicle (DMSO) maintained intact nuclear morphology, while PJLME treated MDA-MB-231cells displayed typical features of apoptosis in which condensed chromatin and pyknotic (shrunken and dark) nuclei were seen (Figure 6). However the cell lines MCF-7 and HaCaT does not showed any apoptotic nuclear signatures when treated with same concentrations of PJLME. The results of the nuclear fragmentation assay clearly demonstrated that the triple-negative MDA-MB-231 cells are more prone for undergoing apoptosis when treated with concerned IC_50_ concentration PJLME.

### Detection of the apoptosis using Annexin-V assay

**Figure 7:**
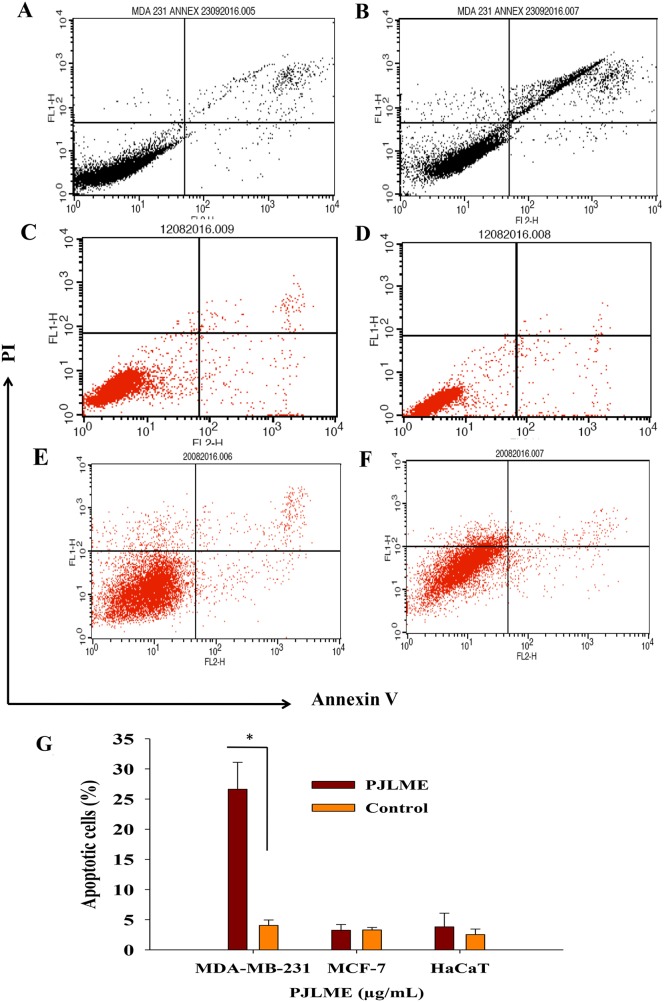
**(A**-**F)** Detection of the PJLME induced apoptosis using Annexin-V assay. Cells were treated with PJLME (16.8μg/mL) and vehicle control (DMSO 0.1%) for 72h. (A) Vehicle treated MDA-MB-231cells, (B) PJLME treated MDA-MB-231 Cells, (C) Vehicle treated MCF-7 cells, (D) PJLME treated MCF-7 cells, (E) Vehicle treated HaCaT cells, and (F) PJLME treated HaCaT cells. **(G)**. Bar diagram showing percentage of apoptotic population after the treatment of PJLME. ^*^values that were significantly different (^*^p < 0.05) from the control.

For re-confirmation of the early apoptosis in MDA-MB-231, MCF-7 breast cancer cells and in non-tumorigenic normal human keratinocytes (HaCaT), the cells were once again treated with the PJLME (16.8 μg/mL) for 72h. After the treatment, cells were analyzed for cell surface specific apoptotic biomarker ‘phosphatidylserine’ by flow cytometry Annexin V-FITC assay. Quantitative analysis of flow cytometry confirmed that treatment of PJLME induced apoptosis only in the MDA-MB-231 cells (26% apoptotic cells) and a very small fraction (3%) was observed in MCF-7 and HaCaT cells (Figure 7A-7F and 7G). Thus, the results of Annexin-V assay reiterated that PJLME specifically induced cell death in the TNBC cell line MDA-MB-231through apoptosis, while the MCF-7 (3% apoptotic cells) breast cancer cell line seems to be less sensitive towards PJLME treatment. Interestingly, the normal HaCaT cell line has very small adverse effect (3% apoptotic cells) as compared to triple negative MDA-MB-231 (26%) cell line.

### Cell cycle analysis

**Figure 8:**
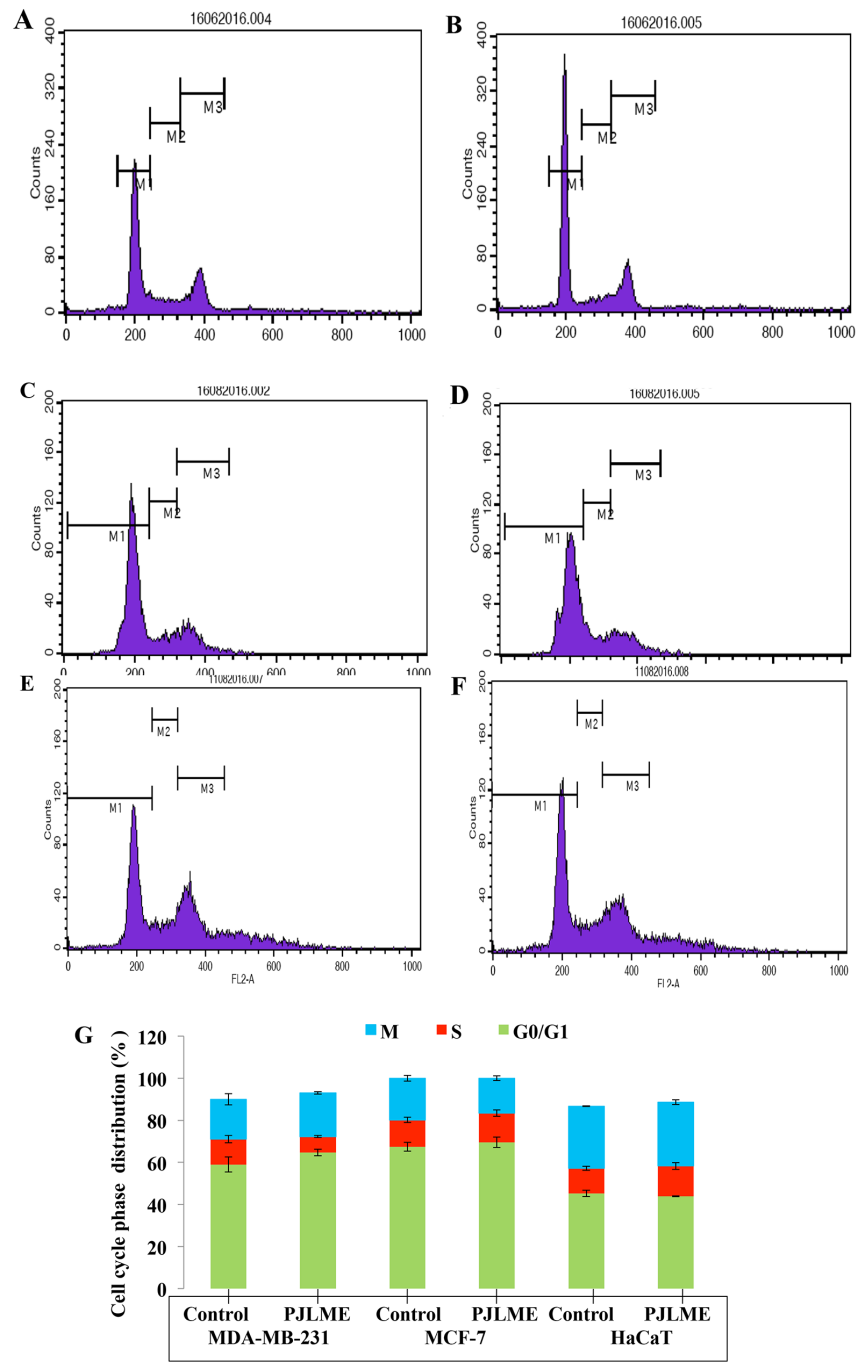
**(A**-**F)** Flow cytometric analysis of effect of PJLME on cell cycle of MDA-MB-231, MCF-7 and HaCaT cells. The cells were treated with PJLME (16.8μg/mL) and vehicle control (DMSO 0.1%) for 72 h & were analyzed using FACS (Calibur flow cytometer; BD Biosciences, CA, USA). The cell cycle phase profile of MDA-MB-231: (A) Control (B) PJLME. The cell cycle phase profile of MCF-7: (C) Control, (D) PJLME. The cell cycle phase profile of HaCaT: (E) Control (F) PJLME treatment. **(G)** Graphical presentation of distribution (%) of cells in different phases of cell cycle. The values in the bars indicates the % population of cells in respective cell cycle phases.

The arrest of cell cycle by the test sample happens to be a rational for developing it as candidate anticancer agent. Previously, variety of plant extracts has demonstrated their cell cycle arresting activities at different phases of cell cycle [21]. To analyze the effect of PJLME on cell cycle arrest at various phases, the selected three cell lines were exposed with the PJLME (16.8 μg/mL) for 72h. After 72h of treatment, the cells were stained with propidium iodide (PI) and the samples were further analyzed using flow cytometry (BD FACS Calibur, USA). The results of the flow cytometry analysis revealed that PJLME treatment with MDA-MB-231 cells resulted in arresting the cell cycle at G0/G1 phase (65%) as compared to control (59%). The population of PJLME treated MDA-MB-231 cells also decreased significantly in the S phase of cell cycle (8%) (Figure 8A-8F & 8G). Of note, HaCaT keratinocytes (44% in G0/G1; 14% in S; 30% in M phase) also escaped from the cell cycle arresting effect of PJLME.

### Inhibition of angiogenesis (CAM assay)

**Figure 9:**
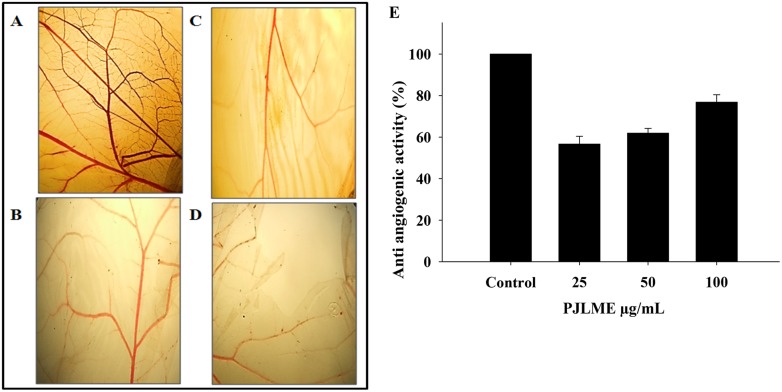
**(A**-**D)** Representative digitized CAMs exposed to different concentrations of PJLME. CAM assay was performed to assess the anti angiogenic properties of the PJLME. (A) Control (PBS), (B) PJLME 25 μg/mL, (C) PJLME 50 μg/mL, (D) PJLME 100 μg/mL. **(E)** Graph showing concentration dependent anti-angiogenic (%) activity of PJLME.

Tumor angiogenesis has been established as one of the hallmark of cancer. The result of the CAM assay summarized in the (Figure 9A-9D & 9E) clearly indicates the dose dependent antiangiogenic potential of PJLME. The results clearly showed that the contents of PJLME possess a significant potential to inhibit the angiogenesis in CAM model.

### Western blot analysis

**Figure 10:**
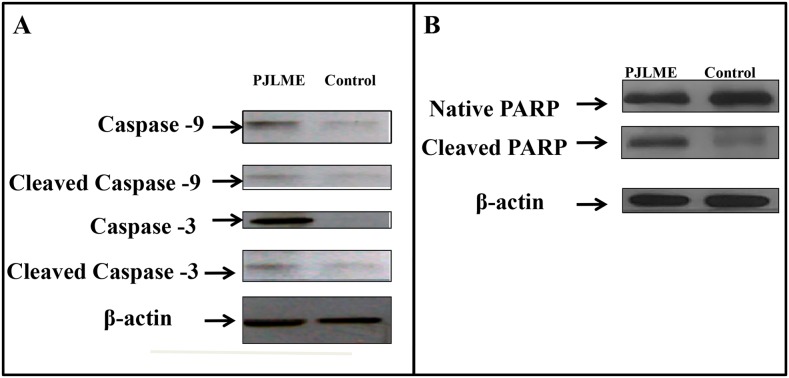
**(A**-**B)** Effect of PJLME on enzymes involved in apoptosis. Profile of apoptosis inducing proteins like caspases 3, 9 and PARP in MDA-MB-231 cells were detected using western blot analysis. MDA-MB-231 cells were treated with PJLME (16.8μg/mL) and control (DMSO 0.1%) for 72h and the cell lysates were subjected to the western blot analysis for assessing the cleavage of caspases 3, 9and PARP enzyme. β actin was used as a loading control. (A) Immunoblotting of caspases 3 and 9 (B) Immunoblotting of PARP in MDA-MB-231 treated and control cells.

Owing to non-significant effects of PJLME on MCF-7 and normal HaCaT cells in majority of the *in vitro* assays carried out, we have performed western blot analysis for detection of the caspases and poly (ADP-ribose) polymerase (PARP) only with PJLME treated MDA-MB-231cells and compared with vehicle control (DMSO). The results of western blot analysis are shown in (Figure 10A & 10B), wherein there is clear indication of down regulation of pro-caspase -9 and -3 and subsequent increasing levels of cleaved active forms of caspase-9 and -3 along with successful cleavage of PARP enzyme.

### *In vivo* anti-breast cancer activity of PJLME using BALB/c mice model

**Figure 11:**
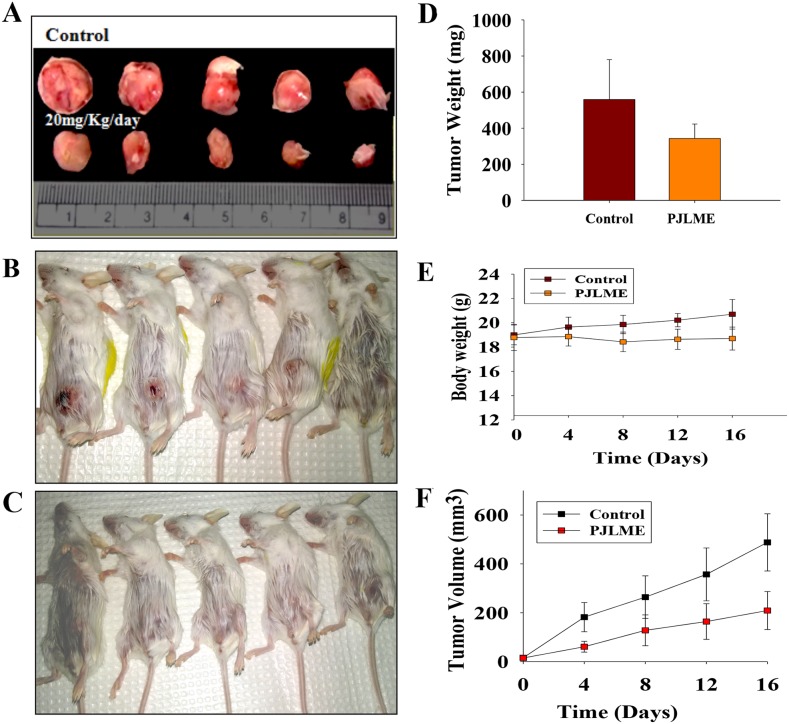
**(A**-**F)** *In vivo* anti-breast activity of PJLME using BALB/c mice model. (A) Representative images of tumors isolated from PJLME treated and untreated (control) mice after 17 days of treatment, (B) Images of Group-1 (control) experimental mice before isolating the tumors, (C) Images of Group-2 (PJLME treated) experimental mice before isolating the tumors, (D) Graph showing the average weight of control and PJLME treated mouse derived tumors, (E) Graph showing body weights of PJLME treated and control mice, (F) Graph showing volume of the isolated tumors from PJLME treated and control mice.

*In vivo* animal studies were conducted in accordance with prior approval of the Institutional Animal Ethics Committee (IAEC), National Centre for Cell Science (NCCS), Pune, MS, India (Approval No. IAEC/2016/B275). *In vivo* anti-breast cancer activity of PJLME was performed using breast cancer tumor xenografts in BALB/c mice. We have used 4T1 syngeneic mice model to evaluate the *in vivo* anti-breast cancer activity of PJLME, as this model has been described as a better suit for mimicking human breast cancer in an immune-competent condition. In brief, female BALB/c mice were divided into 2 groups (*n* = 5), 1^st^ group of mice comprised of untreated control group; while 2^nd^ group of mice was treated (intraperitoneally) with PJLME (20 mg/kg/day) for 17 days. Mice weights and tumor volumes were measured after the sacrifice of animals. The results of the tumor xenograft model studies are summarized in (Figure 11A-11F), which clearly demonstrated the dramatic anti-breast cancer effect of PJLME after 17 days of treatment. The PJLME treatment had a remarkable impact on reducing the size and weight of the 4T1 induced tumors in BALB/c mice when compared with untreated tumors. Of note, the PJLME treatment slightly reduced the weights of the treated mice.

### Histopathological analysis of tumor xenografts

**Figure 12:**
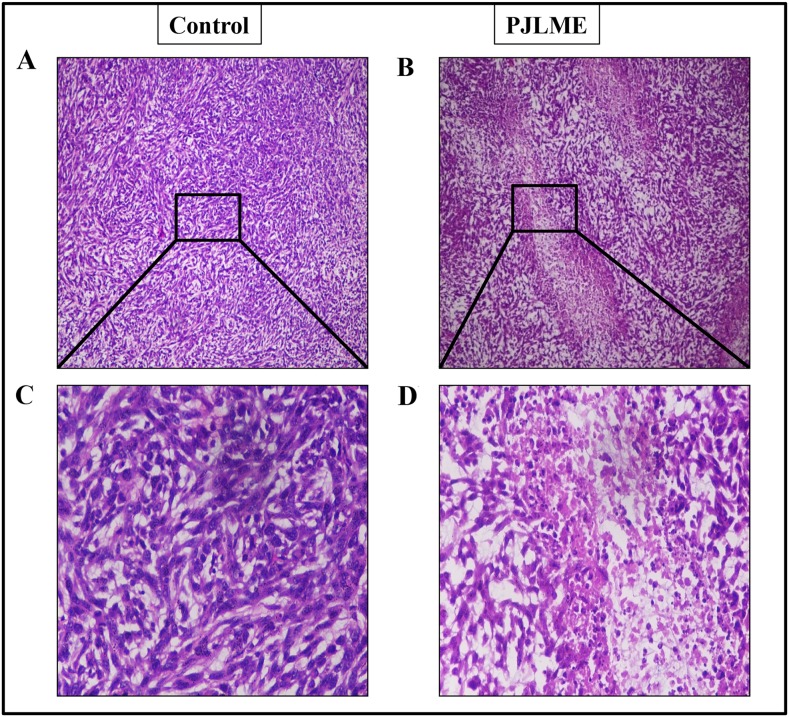
**(A**-**D)** Representative images of the histopathological analysis of PJLME treated and control tumors isolated from the mice. (A) and (C), histopathological images of tumors from Group-1 (control) mice at 10x and 40x magnification respectively, (B) and (D) histopathological images of tumors from Group-2 (PJLME 20mg/Kg/day) mice.

For understanding the intratumor efficacy of PJLME, the tumor xenografts were subjected for histopathological analysis (H & E staining). The results summarized in (Figure 12A-12D) clearly revealed that the PJLME treated tumors were observed to be associated with cell shrinkage, nuclear pyknosis and intratumor spaces owing to death of cells etc.; however the untreated tumors retained normal cell size and nuclear organization to a greater extent.

## DISCUSSION

In the current state of the art the cancer is being studied as an evolving ecosystem. On the eve of emerging drug resistance, the efficacy of the current cancer chemotherapy drugs in general and breast cancer in particular is decreasing with evolving tumor heterogeneity and drug resistance [22]. Moreover, the classical cancer drug discovery process mostly involves processes like purification, synthesis, and development of ‘a single drug for a single target’ [23]. It has been largely observed that the target specificity of a single drug molecule is mostly not specific towards cancer cells; it equally targets normal cells as well and thereby results into severe toxicity to the normal counterpart. Nevertheless, the drugs developed in line with ‘one drug-one target’ may not combat the evolving multifactorial and dynamic tumorigenic processes like cancer heterogeneity and drug resistance [23, 24]. Therefore, in the midst of ‘Target rich-Lead Poor’ scenario of anticancer drug development, the plant extracts/fractions are coming up as a significant hope for development of potent anti-tumor agents, which perhaps may selectively target tumor cells in numerous ways and might prove an effective agent for overcoming emerging drug resistance, mitigating the toxic side effects and acting as an alternative or complementary anti-cancer drug candidates.

The basic aim of the present investigation was to assess the anti-breast cancer potential of PJLME against the hormone independent TNBC (MDA-MB-231 cells) and hormone dependent (MCF-7) breast cancer cells and the normal human origin HaCaT cells. We have conducted series of *in vitro* assays like MTT cell viability/proliferation, effect on cell morphology, cell migration, colony formation, nuclear fragmentation, apoptosis, cell cycle analysis, ROS generation, inhibition of angiogenesis etc. for evaluating the effect of PJLME against the selected MDA-MB-231, MCF-7 breast cancer and normal HaCaT cells. The results of the majority of the assays (summarized in results) clearly demonstrate the remarkable sensitivity of MDA-MB-231cells towards the treatment of PJLME as compared to MCF-7. It is interesting and worth of noting that in all the *in vitro* assays conducted, the normal HaCaT cells had a very small adverse effect of PJLME treatment as compared to breast cancer cells. To describe few, the IC_50_ values calculated in the MTT cell viability /proliferation assay clearly showed that the PJLME treatment was more effective against MDA-MB-231 cells (IC_50_ 16.8 μg/mL) as compared to treatment with MCF-7 cells (19.4 μg/mL). Interestingly, normal HaCaT cells were found to be less sensitive towards PJLME treatment (IC_50_ 24.1 μg/mL). Almost similar trend of activities were observed with other assays like effect on cell morphology, colony formation and cell migration.

Cell cycle analysis is usually employed to distinguish cells in different phases of cell cycle. In this assay, the cells are stained with propidium iodide (PI) owing to its ability to bind DNA and emit fluorescence in UV light; thereby the cellular DNA can be quantified and correlated with different phases of cell cycle. The results of the present investigation has demonstrated that PJLME arrest the cell cycle of MDA-MB-231 at G0/G1 phase more effectively as compared to the cell cycle arrest in MCF-7 and interestingly in normal HaCaT cells. It has been reported that the drug doxorubicin which is widely prescribed for the treatment of breast cancer, it arrest the cell cycle at G2/M phase in MDA-MB-231 cells [25], while the PJLME induces cell cycle arrest at G0/G1 phase, and thereby inhibit the intracellular signaling leading to DNA synthesis and G2/M mitosis. These findings focus the significance of PJLME as a promising source of novel lead/s for arresting cell cycle in breast cancer cells.

Owing to the impressive and selective activity of PJLME against MDA-MB-231 cells, we inspired to design the experiments for unraveling the mechanism of action of PJLME in arresting the growth of selected cancer cells. Sizable volume of literature has accumulated in the recent past linking the role of phytochemicals in inducing the ROS generation followed by apoptosis via intrinsic or extrinsic pathways involving caspases and/or p53-dependent or independent mechanisms [26]. In concert with the present state-of-the-art literature, we have performed the detection of PJLME induced ROS, nuclear fragmentation and finally detection of the apoptosis using Annexin-V assay. The results of the these assays (Figure 5, 6, 7) performed to understand the mechanism of action of PJLME, revealed the fact that PJLME induces ROS generation, nuclear fragmentation (a signature of apoptosis) and induces apoptosis more selectively in MDA-MB-231 cells (26%) as compared to 3% apoptotic cell population in MCF-7 and normal HaCaT cell line. As a part of investigating the role of PJLME in activating the intrinsic apoptosis pathways, we have performed WBA and confirmed activation of the intrinsic apoptosis pathway by detecting cleaved caspase -9 (caspase -9 is the apoptotic initiator protease of the intrinsic or mitochondrial apoptotic pathway) and caspase -3. Caspases are the members of cysteine proteases family, natively expressed as inactive enzymes and have a vital role in apoptosis. Procaspase-9 responds to the release of cytochrome C from mitochondria and interacts with APAF-1(Apoptotic Peptidase Activating Factor -1) and gets activated to caspase-9, resulting in the activation of caspase-3. This activated caspase-3 proteolytically degrades the PARP and fails to repair intracellular damaged DNA and thus completes the intrinsic pathway of apoptosis. The results of the WBA (Figure 10A and 10B) clearly demonstrated the up regulation of caspase-9 and -3 along with successful cleavage of PARP and thereby ruling out the possibility of DNA repair. Inhibition of tumor angiogenesis and related pro-angiogenic cytokines has been identified as one of the important targets for design and development of novel anti-tumor agents. Moreover, currently over ten anti-angiogenic drugs have been approved by the FDA of USA [27]. Therefore the anti-cancer agents inhibiting angiogenesis can be considered as possible drug candidates for the management of cancer. PJLME also demonstrated dose dependent effect in inhibiting angiogenesis in CAM model.

The reliability of *in vitro* assays carried out to address the various issues related to anti-breast cancer activities should be confirmed using *in vivo* animal model studies. Nevertheless, the efficacy of any anti-cancer drug which intended for anti-tumor activity should be active in the *in vivo* condition. We have used 4T1cells (mice derived TNBCs) syngeneic BALB/c mice model to evaluate the *in vivo* anti-breast cancer activity of PJLME as this model happens to be a better suited model for mimicking human breast cancer in an immune-competent condition. The results of the *in vivo* studies of the PJLME clearly demonstrated the impressive *in vivo* anti-breast cancer activity in BALB/c mice. PJLME treatment had a remarkable impact on reducing the size and weight of the 4T1 induced tumors in BALB/c mice when compared with untreated tumors. According to the United States National Cancer Institute (US-NCI) criteria for crude extract, it has been described that the extracts which shows an IC_50_ values less than 100 μg/mL can be considered as an active sample, while the crude sample having IC_50_ less than 30 μg/mL, can be considered promising sample for further purification of bioactive compounds [28]. As per the NCI guidelines, the PJLME can be considered as a promising extract as the IC_50_ values calculated against MDA-MB-231 (IC_50_16.8 μg/mL) and MCF-7 (19.4 μg/mL) breast cancer cells are less than the limit (30 μg/mL) described by NCI. The GC-MS chemo profile of *P. juliflora* are available in the literature and it has been described to possess array of bioactive compounds belonging to phenolics, flavonoids, alkaloids, terpenes, steroids and tannins [29].

Moreover, as a part of quality control of the sample, we have also analyzed the phytochemicals of PJLME using GCMS-TQ8030 Triple Quadrupole Gas Chromatograph Mass Spectrometer (Shimadzu, Japan) and detected variety of phytochemicals (Table 1) including phthalic acid and its variety of esters like ethyl isoporpyl ester, 5-methylhex-2-yl ethyl ester, 8-chlorooctyl ethyl ester, ethyl pentyl ester, 2-chloropropyl ethyl ester, ethyl 2-(2-nitrophenyl) ethyl ester, 5-methylhex-2-yl ethyl ester, 2-acethylphenyl ethyl ester, ethyl 3-ethylphenyl ester. The other notable phyto constituents includes myo-Inositol (4-C-methyl), l-(+)-ascorbic acid 2,6-dihexadecanoate, phytol (2-hexadecen-1-ol), isophytol acetate and cyclohexanol (5-methyl-2-(1-methylethyl). The *in vitro* and *in vivo* anti-breast cancer activities of PJLME can be attributed with the aforesaid bioactive molecules as plethora of literature has been cited towards the anti-cancer potential of these groups of phytochemicals [26].

**Table 1:** Chemical structures of compounds identified in PJMLE using GC-MS

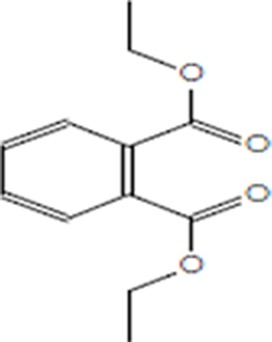 1,2-Benzenedicarboxylic acid (diethyl Phthalate)	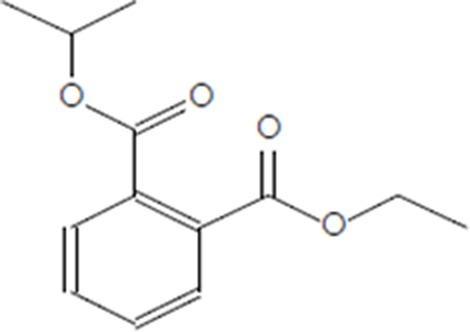 Ethyl isoporpyl ester of phthalic acid	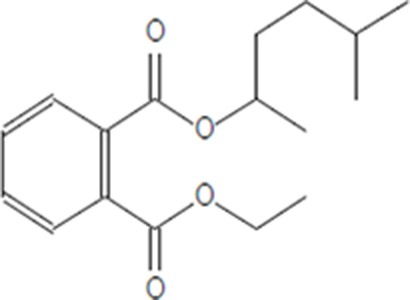 5-methylhex-2-yl ethyl ester of phthalic acid
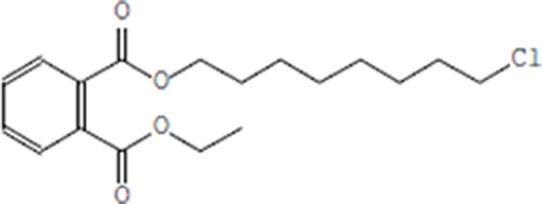 8-chlorooctyl ethyl ester of phthalic acid	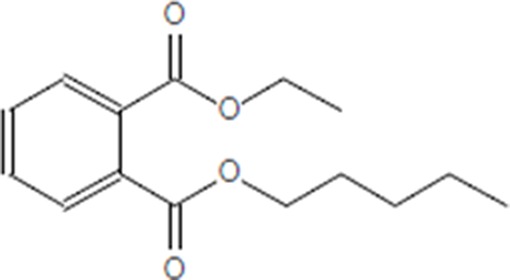 Ethyl pentyl ester of phthalic acid	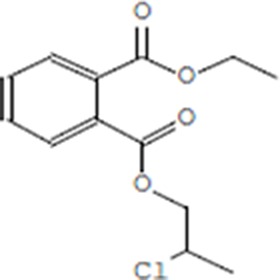 2-chloropropyl ethyl ester of phthalic acid
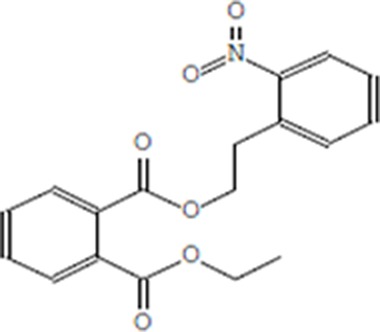 Ethyl 2-(2-nitrophenyl) ethyl ester of phthalic acid	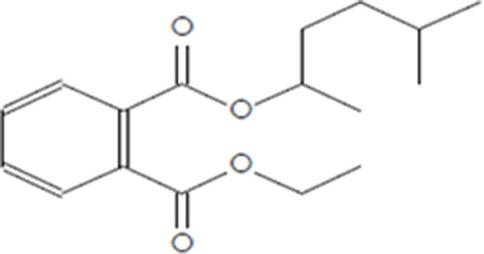 5-methylhex-2-yl ethyl ester of phthalic acid	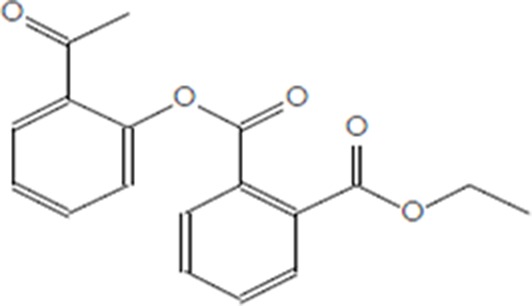 2-acethylphenyl ethyl ester of >phthalic acid
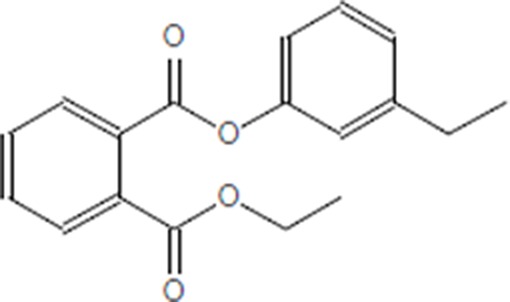 Ethyl 3-ethylphenyl ester of phthalic acid	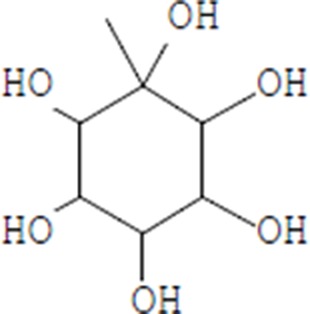 Myo-Inositol (4-C-methyl)	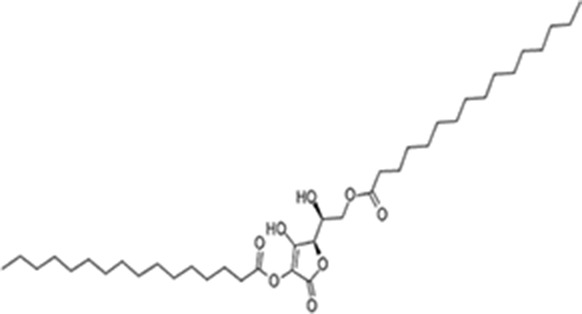 l-(+)-Ascorbic acid 2,6-dihexadecanoate
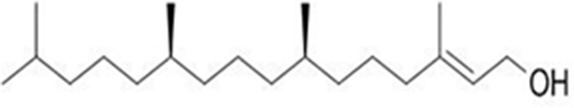 Phytol (2-Hexadecen-1-ol)	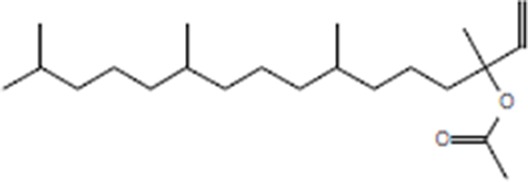 Isophytol, acetate	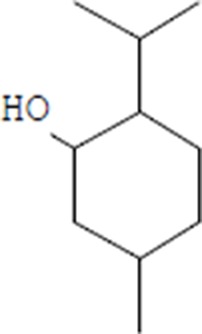 Cyclohexanol (5-methyl-2-(1-methylethyl)-

For example, series of phthalic acid derivative polymers with medium-molecular-weight have been reported to possess antitumor and anti-angiogenic activities [30]. Bizzarri et al., have recently reviewed the broad spectrum anticancer activities of myo-inositol in both physiological and pathological settings [31]. In an *in vitro* experimental settings, phytol has been demonstrated to induce apoptosis in human gastric adenocarcinoma AGS cells, downregulates Bcl-2, upregulates Bax, activates caspase-9 and -3 and induces PARP cleavage [32]. Besides consistent reports of cancer cell selective toxicity induced by high-dose of ascorbic acid treatment in preclinical settings, the mechanism of anticancer effect of ascorbic acid has remained elusive and the research in this regard is evolving [33].

## MATERIALS AND METHODS

### Chemicals, media and reagents

Cell culture media such as DMEM/F12, Leibovitz's (L-15), Eagle's Minimum Essential Medium (EMEM), Sodium pyruvate, Fatal Bovine Serum (FBS), Dimethyl sulfoxide (DMSO), 3-(4, 5-dimethylthiazolyl-2)-2, 5-diphenyl tetrazolium bromide (MTT), Propidium iodide (PI), 2`7`-2′,7′-Dichlorodihydrofluorescein diacetate (DCFH-DA), Annexin FITC/PI kit, RNase A, Hoechst 33258, were purchased from Sigma Chemical Co. (St. Louis, MO, USA). Seablue Protein Ladder was obtained from Thermo Fisher Pvt. Ltd. Anti caspase-3 (cleaved) and anti caspase-9 (cleaved) were obtained from Cell Signaling Technology, β-Actin antibody and anti PARP antibody purchased from Santa Cruz Biotechnology. All other chemicals and reagents were of AR grade and were purchased from commercial sources.

### Collection of the plant material, preparation of test sample and phytochemical analysis using GC-MS

Fresh leaves of the *Prosopis juliflora* (Sw.), DC. were collected from the campus of Swami Ramanand Teerth Marathwada University (SRTMU), Nanded (MS), India. The botanical identity of the plant specimen of *P. juliflora* was confirmed by Dr. R. M. Mulani, taxonomist at Department of Botany, SRTM University, Nanded (MS), India and it was authenticated to be *Prosopis juliflora* (Sw.), DC belonging to a family of Mimosaceae. The voucher specimen of the collected plant was deposited in the Department of Botany of S.R.T.M. University, Nanded (MS). The collected fresh leaves of *P. juliflora* were initially washed with tap water and finally with distilled water. Clean leaves were shade dried and was made into fine powder. The powdered plant sample was extracted in methanol using Soxhlet's extraction system up to 8 hours at 60^°^C. The obtained methanol extract was evaporated under reduced pressure at approximately 40°C and stored in the sterile tubes at -20^°^C until the experiments.

The phytochemical analysis of the PJLME was carried out using GCMS-TQ8030 Triple Quadrupole Gas Chromatograph Mass Spectrometer analysis system (Shimadzu, Japan) equipped with a Rtx-5MS (5% diphenyl/95% dimethyl polysiloxane) silica fused capillary column (30m × 0.25mm I.D. × 0.25μm df). For GC-MS analysis an electron ionization system was operated in electron impact mode with an ionization energy of 70 eV. Helium gas (99.99%) was used as a carrier gas at a constant flow rate of 1.20 mL/min, and an injection volume of 1 μL was employed (a split ratio of 40:1). The injector temperature was maintained at 250°C, the ion-source temperature was 230°C, the oven temperature was programmed from 50°C (isothermal for 2 min), with an increase of 10°C/min to 240°C, then 5°C/min to 280°C, ending with a 9 min isothermal at 280°C. Mass spectra were recorded at a scan interval of 0.3 s. The solvent delay was 0 to 3 min, and the total GC-MS running time was 34 min. The relative percentage amount of each component was calculated by comparing its average peak area to the total areas.

### Cell cultures

For assessing anti-breast cancer activity of PJLME, we have selected MDA-MB-231 cells tumorigenic, invasive and highly metastatic as a representative of TNBC (ER^-^, PR^-^, HER2^-^) nature cell line from the human origin and MCF-7 (ER^+^, PR^+/-^) as a representative of hormone dependent breast cancer [34]. The MDA-MB-231 cells were cultured in L-15 (Leibovitz's 15) medium, while MCF-7 cell line was cultured in Eagle's Minimum Essential Medium (EMEM) with 1mM sodium pyruvate. As a representative of normal or non-tumorigenic control, a human keratinocytes cell line HaCaT was used; it was cultured using DMEM with addition of nutrient mixture F-12 Ham in 1:1 ratio [35]. Mouse breast cancer cell line (4T1) was a kind gift from Dr. Wani Mohan, NCCS, Pune (MS), India and was maintained in RPMI 1640 medium. All other cell lines were obtained from National Centre for Cell Science: a national facility for providing animal cell lines, Pune (MS), India. All the cell lines were supplemented with 10% FBS (fetal bovine serum), 50 units/mL penicillin, 50 μg/mL streptomycin and were maintained in a humidified atmosphere with or without 5% CO_2_ at 37^°^C temperature.

### Effect of PJLME on cell proliferation (MTT assay)

*In vitro* cytotoxic effect of PJLME was evaluated against MDA-MB-231, MCF-7 and HaCaT cells. The MTT assay was carried out to assess the cytotoxic effect of PJLME against selected cell lines as previously described with certain modifications [36, 37]. In brief, the individual cells were seeded in the triplicates at a density of 1×10^5^ cells/mL in the 96-well culture plate (Falcon) and after the 24h of incubation; cells were treated with the different concentrations of PJLME for 72h at 37°C in a humidified atmosphere with or without 5% CO_2_. After the treatment, PJLME containing medium was replaced with 50 μL of MTT solution (2 mg/mL) prepared in phosphate-buffered saline (PBS, pH 7.2) and plates were then incubated for 4h at 37^°^C. After the incubation time, the crystals of the formazan were dissolved in 100μL of DMSO and the absorbance of colored formazan product was measured at 570 nm using a microtiter plate reader (Multiskan GO, Thermo Scientific, USA) at 570 nm. The IC_50_ values were calculated as such concentrations of PJLME which demonstrated 50% cytotoxicity against selected individual cell lines. The results were expressed as% cytotoxicity.

### Effect of PJLME on morphology of selected cells

Individual cell lines (5 × 10^4^) were seeded onto glass cover slip in a 12 well plate and incubated at 37^°^C for 24h. After the incubation, cells were treated with the PJLME (16.8μg/mL) and incubated for 72hours at 37°C in a humidified atmosphere with or without 5% CO_2_. After the treatment, the cells were observed and digitized using DP71 camera attached fluorescence microscope (Olympus, Tokyo, Japan) at 10X magnification.

### Cell migration assay

For evaluation of the migration inhibition efficiency of PJLME, a scratch assay was performed on confluent monolayers of the MDA-MB-231 and HaCaT cells. The assay was performed as described in previous report [38] with slight modifications. In brief, the individual cells (2-4 × 10^5^) were seeded in to 24 well plates and kept for 2 days to form a complete monolayer. A scratch was made by using a sterile 200μL pipette tip and after washing with PBS cells were grown in the media containing PJLME (16.8μg/mL). The migration of the cells in the ‘scratch’ area was measured at 0h and 12h, images were captured at 4X magnification using an inverted Nikon microscope equipped with a digital camera and analyzed by using NIH Image J software.

### Effect of PJLME on colony formation (Clonogenic assay)

The clonogenic assay was performed as described earlier [39] with slight modifications. Briefly, breast cancer specific MDA-MB-231, and non-tumorigenic HaCaT cells were seeded in 6 well plates (2 × 10^5^ cells/well) and treated with the PJLME (16.8μg/mL) for 72h. After the treatment, cells were washed with the PBS 1X (pH 7.4) and 2 × 10^3^ cells/well were reseeded in to the 6 well plate with addition of fresh cell culture medium and 10% FBS. Cells were incubated for next 7 or 12 days and medium was replaced after every 3^rd^ day. Colonies were fixed with 4% paraformaldehyde and stained with 0.5% crystal violet. Stained cells in each colony (with more than 50 cells) were determined by phase contrast microscopy and the images were captured. Colony sizes on images were measured using NIH Image J software. The data is presented as mean colony number ± SD relative to untreated controls (n=3 independent experiments).

### Detection of PJLME induced nuclear fragmentation

To assess the effect of PJLME on nuclear integrity, we have performed a nuclear fragmentation assay. In brief, individual cells (5 × 10^4^) growing in the log phase were seeded onto sterile glass cover slips in 12 well plates and incubated for 24h. After the incubation, the cells were treated with the PJLME (16.8 μg/mL) for 72h at the 37°C in a humidified atmosphere with or without 5% CO_2_. After the treatment, cells were fixed in a 4% paraformaldehyde and stained with Hoechst 33258 nuclear stain (5 μg/mL in PBS, pH 7.4) for 10 minutes in a dark chamber at room temperature. Following the staining procedure, cells were washed once with PBS and the cover slips were mounted with a fluorescence mounting medium (Dako, Glostrup, Denmark). The mounted cells were observed under fluorescence microscope and digitized using DP71 camera (Olympus, Tokyo, Japan) at 20X objective. Apoptotic cells were further characterized as cells displaying chromatin condensation or nuclear fragmentation.

### Detection of PJLME induced apoptosis (Annexin V assay)

To detect the PJLME induced apoptosis in PJLME treated cells, we have performed an Annexin V assay. The assay was carried out using ‘Annexin V-FITC Apoptosis Detection Kit’ (Sigma). The experimental protocol followed was as per the manufacturer instructions. In brief, the cell lines were seeded at a density of 2 × 10^5^ in six-well plates and incubated for 24h, after the incubation, the growing cells were treated with the PJLME (16.8 μg/mL) for 72h at the 37°C in a humidified atmosphere with or without 5% CO_2._ Following the treatment, minimum of 10000 of individual stained cells per sample were analyzed by using the flow cytometer (BD FACS Calibur, USA) and results were analyzed using the BD Cell Quest Pro software.

### Studies on detection of PJLME induced intracellular ROS

The effect of PJLME on generation of intracellular ROS was performed as described in previous investigation [40] with slight modifications. In brief, the selected cells (1 × 10^4^) were seeded in 96 well plates and incubated for 24h. After the incubation, cells were treated with the PJLME (16.8 μg/mL) for 72h at the 37°C in a humidified atmosphere with or without 5% CO_2._ After treatment, cells were washed with PBS (pH 7.4) and exposed to DCFH-DA (10 μM) stain for 30 min followed by washing with the PBS. Finally, the cells were observed under fluorescence microscope (Olympus, Tokyo, Japan) for presence of 2′,7′-dichlorofluorescein (DCF). The DCF fluorescence images were captured using DP71 camera attached with fluorescence microscope.

### Effect of PJLME on cell cycle (Flow cytometry analysis)

The PJLME treated breast cancer cells and normal cells were analyzed for its effect on arresting cell cycle at various phases of cell cycle as previously reported [41]. Briefly, the selected cells were seeded at a density of 2 × 10^5^ in 6 well plates and after 24h of incubation growing cells were treated with the PJLME (16.8 μg/mL) for 72h at the 37°C in a humidified atmosphere with or without 5% CO_2._ After the treatment, cells were fixed in 70% ice-cold ethanol at -20^°^C for 4h and stained with propidium iodide (prepared in PBS 1X; 0.1% sodium citrate, 0.1% Triton X-100, 250 μg/mL RNase A, and 50 μg/mL propidium iodide) and DNA contents of stained nuclei were analyzed by using flow cytometer (BD FACS Calibur, USA) and results of the flow cytometer were analyzed using the BD Cell Quest Pro software.

### Effect of PJLME on angiogenesis

The chorioallantoic membrane (CAM) assay was performed as described in our previous reports [42]. The anti-angiogenic potential of PJLME was determined using formula, 1−T/C, where T indicates the no. of blood vessels in the PJLME treated sample, while C indicates the no. of blood vessels present in control (PBS). The control and treated CAMs were digitized using an Olympus make SZ 61TR Zoom Trinocular Microscope attached with CCD camera and an image capturing software Pinnacle v.6.0.2 (build 152). The images of the treated and control CAMs were further subjected for analysis using an image analysis software AngioQuant v 1.33 (a MATLAB-based software tool for quantification of angiogenesis) for the analysis of number, length, size, and the junctions of the tubule complexes (data not shown).

### Western blot analysis

Western blot analysis (WBA) was carried out to understand the expression profile of enzymes caspase 3, 9; PARP and confirm the apoptosis mode of cell attenuation by PJLME. Owing to the less sensitivity of MCF-7 and HaCaT cell lines towards PJLME treatment in various assays, only MDA-MB-231 cell line was considered for WBA. The WBA was carried out as per the previously described method and with some modifications [43]. In brief, 2 × 10^5^ cells seeded in 6 well plates and incubated for next 24h, after the incubation, the growing cells were treated with the PJLME (16.8 μg/mL) for 72h at 37°C in a humidified atmosphere without 5% CO_2._ After treatment, the cells were washed with PBS, scraped, pelleted and lysed in RIPA buffer containing protease inhibitor cocktail (Roche). After the incubation (30 min on ice), the cell lysates were centrifuged at 14,000 rpm for 20 min at 4°C. Cell protein concentration was determined by bradford protein assay (BioRad). Total cell lysate protein (25 μg) were resolved onto 8-12% SDS-PAGE along with Seablue Protein Ladder.

### Study of *in vivo* anti-breast cancer activity of PJLME

#### Experimental animals

The animal experimental protocol of the study was approved by the Institutional Animal Ethics Committee, of National Centre for Cell Science, Pune (MS), India (Approval No. IAEC/2016/B275). Experiments were performed on normal (immune competent) female BALB/c mice with 20-30 g in weight and age of 6-8 weeks. The animals were housed individually in cages under standard laboratory conditions with a period of 12/12-h light/dark cycle, below 30°C with 40–50% relative humidity. The mice were freely allowed to drink water and eat standard chow pellets.

#### Experimental design

Ten BALB/c female mice were divided into 2 groups (*n* = 5); Group-1 was with 4T1 induced breast cancer and control; while Group-2 was with 4T1 induced breast cancer and treated with PJLME (20 mg/kg/day). All the animals in group 1 and 2 were injected with 4T1 cells (1 × 10^5^/0.1 mL of PBS, pH 7.2) into the mice mammary fat pad. After getting palpable tumor, PJLME was administered intraperitoneally (IP) to the Group-2 animals for 17 days. The mice weight and tumor volume was recorded after every 4^th^ day. Animals were sacrificed on 17^th^ day of treatment and the weight of the treated and control animals was recorded. The volume (V) of the tumor was calculated using a formula; V = L × W^2^ × 0.52 (L-length, W-width).

### Statistical analysis

All the *in vitro* experiments carried out in triplicates. The data presented are the means ± S.D, and statistical differences between various treatments were analyzed by using Student's *t*-test.

## CONCLUSIONS

As per our knowledge, this is a first kind of report describing the *in vitro* and *in vivo* efficacy of PJLME against hormone dependent and independent breast cancer cells. The results of both settings clearly focus the significance of the PJLME as an attractive agent against the breast cancer in general and TNBCs in particular. One of the important aspect of the present investigation is that the PJLME is more selective towards inhibition of TNBCs like MDA-MB-231 as compared to hormone dependent MCF-7 breast cancer cells and interestingly it has very small adverse effect against normal human keratinocytes like HaCaT cells. Results of the present study have established the importance of *P. juliflora* as a potential resource of metabolites which could be further explored for the development of novel anti-cancer agents targeting breast cancer. The results may also act as a ready reference towards developing *P. juliflora* based formulation as an alternative and complementary medicine for the management of the breast cancer. Further bioactivity guided fractionation of different parts of PJLME for isolation and purification of active constituents is in progress.

## Abbreviations

PJLME: *Prosopis juliflora* leaves methanol extract
TNBC: triple negative breast cancer
MTT: 3-(4
PARP: poly (ADP-ribose) polymerase
DCFH-DA: 2′
ROS: reactive Oxygen Species
WBA: Western blot analysis
FBS: fetal bovine serum
IC_50_: half maximal inhibitory concentration.
