# Correction: *Prosopis juliflora* (Sw.), DC induces apoptosis and cell cycle arrest in triple negative breast cancer cells: *in vitro* and *in vivo* investigations

**DOI:** 10.18632/oncotarget.26095

**Published:** 2018-08-31

**Authors:** Bhimashankar Gurushidhappa Utage, Milind Shivajirao Patole, Punam Vasudeo Nagvenkar, Sonali Shankar Kamble, Rajesh Nivarti Gacche

**Affiliations:** ^1^ National Centre for Cell Science, NCCS Complex, Pune, 411007, MS, India; ^2^ School of Life Sciences, S.R.T.M. University, Nanded, 4316069, MS, India; ^3^ Department of Biotechnology, Savitribai Phule Pune University, Pune, 411007, MS, India

**This article has been corrected:** The correct order of authors' names is given below:

**Bhimashankar Gurushidhappa Utage^1,2^, Milind Shivajirao Patole^1^, Punam Vasudeo Nagvenkar^1^, Sonali Shankar Kamble^2^ and Rajesh Nivarti Gacche^2,3^**

Original article: Oncotarget. 2018; 9:30304-30323. https://doi.org/10.18632/oncotarget.25717

